# Aryl Hydrocarbon Receptor and Cysteine Redox Dynamics Underlie (Mal)adaptive Mechanisms to Chronic Intermittent Hypoxia in Kidney Cortex

**DOI:** 10.3390/antiox10091484

**Published:** 2021-09-17

**Authors:** Maria João Correia, António B. Pimpão, Filipa Lopes-Coelho, Catarina O. Sequeira, Nuno R. Coelho, Clara Gonçalves-Dias, Robert Barouki, Xavier Coumoul, Jacinta Serpa, Judit Morello, Emília C. Monteiro, Sofia A. Pereira

**Affiliations:** 1 CEDOC, NOVA Medical School, Universidade Nova de Lisboa, 1169-056 Lisboa, Portugal; mjoao.correia@nms.unl.pt (M.J.C.); antonio.pimpao@nms.unl.pt (A.B.P.); filipa.coelho@nms.unl.pt (F.L.-C.); catarina.sequeira@nms.unl.pt (C.O.S.); nuno.coelho@nms.unl.pt (N.R.C.); clara.dias@nms.unl.pt (C.G.-D.); jacinta.serpa@nms.unl.pt (J.S.); judit.morello@nms.unl.pt (J.M.); emilia.monteiro@nms.unl.pt (E.C.M.); 2 Instituto Português de Oncologia de Lisboa Francisco Gentil (IPOLFG), Rua Prof Lima Basto, 1099-023 Lisboa, Portugal; 3 INSERM UMR-S 1124, 3TS, Environmental Toxicity, Therapeutic Targets, Cellular Signaling and Biomarkers, Université de Paris, 45 rue des Saints-Pères, 75006 Paris, France; robert.barouki@parisdescartes.fr (R.B.); xavier.coumoul@parisdescartes.fr (X.C.)

**Keywords:** thiols, non-radical oxidative species, obstructive sleep apnea, arterial hypertension, CYP1A1, xCT, cystine, precision medicine, animal models, endothelial dysfunction

## Abstract

We hypothesized that an interplay between aryl hydrocarbon receptor (AhR) and cysteine-related thiolome at the kidney cortex underlies the mechanisms of (mal)adaptation to chronic intermittent hypoxia (CIH), promoting arterial hypertension (HTN). Using a rat model of CIH-HTN, we investigated the impact of short-term (1 and 7 days), mid-term (14 and 21 days, pre-HTN), and long-term intermittent hypoxia (IH) (up to 60 days, established HTN) on CYP1A1 protein level (a sensitive hallmark of AhR activation) and cysteine-related thiol pools. We found that acute and chronic IH had opposite effects on CYP1A1 and the thiolome. While short-term IH decreased CYP1A1 and increased protein-*S*-thiolation, long-term IH increased CYP1A1 and free oxidized cysteine. In addition, an in vitro administration of cystine, but not cysteine, to human endothelial cells increased *Cyp1a1* expression, supporting cystine as a putative AhR activator. This study supports CYP1A1 as a biomarker of obstructive sleep apnea (OSA) severity and oxidized pools of cysteine as risk indicator of OSA-HTN. This work contributes to a better understanding of the mechanisms underlying the phenotype of OSA-HTN, mimicked by this model, which is in line with precision medicine challenges in OSA.

## 1. Introduction

Obstructive sleep apnea (OSA) is the most common sleep-related breathing disorder, and its prevalence has risen over time [1]. This clinical condition is characterized by repetitive episodes of apneas (airflow cessation) or hypopneas (airflow reduction) due to the collapse of the upper airway (either partial or complete) during sleep. These stops in breathing promote arousals with sleep fragmentation, hypercapnia, increased intrathoracic pressure, and intermittent hypoxia (IH). Chronic intermittent hypoxia (CIH) associated with OSA has been recognized as responsible for most OSA-related comorbidities, namely systemic hypertension (HTN) [2].

CIH involves desaturation/reoxygenation cycles resembling the pathophysiological mechanisms of ischemia/reperfusion in multi-organ injury, particularly in the kidney. It has been proposed that CIH cycles increase reactive oxygen species (ROS) [3], which are associated with the stabilization of hypoxia-inducible factors (HIFs) [4] and inflammation in renal tissue [5,6,7,8]. However, several clinical trials have shown a disappointing efficacy of the use of free radical scavengers in cardiovascular disease [9,10]. This might support research in novel antioxidant mechanisms of action, namely targeting non-free radical species such as thiol disulfides, e.g., cystine [11]. In fact, we have previously shown that CIH changes the dynamics of cysteine pool to an oxidative status in the kidney when HTN is established [7]. Cysteine (Cys) is the predominating thiol in extracellular fluids [12,13] and its availability is a net contribution of three pools which can dynamically exchange one-to-one depending on the redox status of the tissue involved [7,14,15,16]. The protein-bound fraction (*S*-cysteinylated proteins, CysSSP) is generated by a reversible post-translational modification through a disulfide bond [14]. The free cysteine pool includes the reduced (CysSH) and oxidized cysteine fractions (CysSSX). The oxidized free cysteine pool contains mixed disulfides, like cysteine-glutathione disulfide, but it is majorly composed by cystine (the combination of two cysteines), which can be used as a biomarker of oxidative stress [17,18].

Besides cysteine, the oxidative dynamics of other thiols, such as glutathione (GSH) or cysteinylglycine (CysGly), represent discrete redox pathways, whose disruption might be the origin of oxidative stress [19]. Importantly, these redox pathways vary independently and their levels are tissue-dependent [20].

Aryl hydrocarbon receptor (AhR) is a ligand-activated transcription factor that belongs to the basic helix-loop-helix/Per-Arnt-Sim (bHLH/PAS) family [21,22]. AhR was initially known to be activated by some environmental pollutants such as aryl hydrocarbons like dioxins and benzo[a]pyrene. Nonetheless, as the knowledge concerning the AhR field evolved, it unveiled its implication in relevant and diverse physiological processes, such as the control of the cell cycle, the development of the immune, renal, or cardiovascular systems, and oxidative balance [23,24]. In the canonical pathway and upon the binding of a ligand, the AhR is translocated to the nucleus, dimerizes with AhR nuclear translocator (ARNT), and promotes the transactivation of its target genes, namely CYP1A1. This gene is the hallmark of AhR activation [25,26,27]. Alternatively, AhR may also interact with other pathways, some of them relevant in the context of the oxidative homeostasis, like the nuclear factor erythroid 2-related factor 2 (NRF2) [22,28]. Additionally, AhR ligands such as benzo[a]pyrene and 2,3,7,8-tetrachlorodibenzodioxin (TCDD) promote ROS generation, namely by CYP1 isoforms. The interplay with the NRF2 pathway is known to regulate the expression and activation of antioxidant genes including NAD(P)H:quinone oxidoreductase 1 (NQO1) and superoxide dismutase 1 (SOD1) [28,29].

We recently proposed the existence of an AhR-related phenotype of HTN [27] and proved that the AhR antagonist CH-223191 was able to revert CIH-increased blood pressure (BP) [26]. Using a model of CIH-induced HTN, we reported the activation of the AhR canonical pathway in the kidney cortex [26] at established HTN, as ascertained by an increased expression of the AhR target gene CYP1A1.

Although it is still unclear how CIH activates AhR, we are pursuing the hypothesis that it can be related to Cys oxidation, as the kidney is an organ with high Cys availability [30] and, like AhR, cysteine/cystine redox signaling is an important player in cardiovascular health [7,26,27,31]. Herein, we investigated this interplay, providing novel evidence on the impact of IH chronicity on AhR activation and Cys oxidative dynamics (in vivo), besides linking cystine as an AhR activator in endothelial cells.

## 2. Materials and Methods

### 2.1. Reagents

For Western-blot, the following reagents were used: Anti-β-actin antibody (8H10D10, Cell Signaling Technology, Leiden, The Netherlands); Anti-CYP1A1 antibody (E-AB-13483, ElabScience, Houston, TX, USA); Anti-mouse antibody (SC-516102, Santa Cruz Biotechnology, Dallas, TX, USA); Anti-rabbit antibody (SC-2357, Santa Cruz Biotechnology); BCA Protein Assay kit (23225, ThermoScientific, Waltham, MA, USA); Bovine serum albumin (BSA) (P6154, Biowest SAS, Nuaillé, France); ECL kit (RPN2232, GE Healthcare, Munich, Germany); Ethylenediamine tetraacetic acid (EDTA) (E9884, Sigma Aldrich, St. Louis, MO, USA); Nitrocellulose membranes (10600004, GE Healthcare); Protease inhibitors (04693159001, Roche Diagnostics, Grenzach, Germany); Protein marker (A8889, ITW reagents, Chicago, IL, USA); Sodium fluoride (NaF) (201154, Sigma Aldrich); Sodium orthovanadate (Na_3_VO_4_) (S6508, Sigma Aldrich); Sodium pyrophosphate (Na_4_P_2_O_7_) (P8010, Sigma Aldrich); Tris-base (BP152-1, Fisher Scientific, Waltham, MA, USA); Triton (T92874, Sigma Aldrich).

For thiol, creatinine and albumin sample-pretreatment and quantification the following reagents were used: 7-fluorobenz-2,1,3-oxadiazole-4-sulfonic acid ammonium salt (SBD-F), (46640, Sigma Aldrich); Bromocresol Green Albumin (BCG) Assay Kit (MAK124, Sigma-Aldrich); Creatinine (Cr) (C4255-10G, Sigma-Aldrich); Ethylenediaminetetraacetic acid (EDTA) (E9884, Sigma Aldrich); L-Cysteine (Cys) (30089, Sigma Aldrich); L-Cysteine-L-Glycine (CysGly) (C0166, Sigma Aldrich); L-Glutathione reduced (GSH) (G6529, Sigma Aldrich); Methanol (20864.320, VWR Chemicals, Radnor, PA, USA); Phosphate buffered saline (PBS1X) (P4417, Sigma Aldrich); Potassium dihydrogen phosphate (1.04873.1000, Merck, Darmstadt, Germany); Sodium acetate (27653.292, VWR Chemicals); Sodium hydroxide (NaOH) (30620-M, Sigma Aldrich); Sodium tetraborate decahydrate buffer (Na₂B₄O₇·10H₂O) (27727.231, VWR Chemicals); Trichloroacetic acid (TCA) (8789.1, Carl Roth, Karlsruhe, Germany); Tris(2-carboxyethyl)phosphine hydrochloride (TCEP) (75259, Sigma Aldrich).

### 2.2. Animals

Male Wistar rats Crl:WI (Han) (*Rattus norvegicus* L.) were obtained from the NOVA Medical School animal facility. The use of male rats is justified by the higher prevalence of OSA in men [32]. The rats were housed two per cage in polycarbonate cages with wire lids (Tecniplast, Buguggiate, Varese, Italy) and maintained under standard laboratory conditions: artificial 12 h light/dark cycles (9 a.m. to 9 p.m.), at room temperature (22 ± 2.0 °C) and a relative humidity of 60 ± 10%. The rats were maintained on a standard laboratory diet (SDS diets RM1, Special Diets Services) and reverse osmosis water ad libitum. Corncob bedding (Probiológica, Lisbon, Portugal) was used and changed weekly. The animals were specific-pathogen-free, according to FELASA recommendations [33]. The 3Rs policy was employed to minimize the number of animals used.

All applicable institutional and governmental regulations concerning the ethical use of animals were followed: the NIH Principles of Laboratory Animal Care (NIH Publication 85-23, revised 1985), the European guidelines for the protection of animals used for scientific purposes (European Union Directive 2010/63/EU), and the Portuguese Law nº 113/2013. The Ethical Committee of NOVA Medical School approved all the experimental procedures (protocol nº 15/2017/CEFCM).

#### 2.2.1. Study Design

A total of 69 rats were randomly assigned and divided into 11 groups according to the submission to intermittent hypoxia or normoxic conditions and the respective duration of 1, 7, 14, 21, 35, and 60 days. The sample size for short-term CIH (normotension) period was five animals for groups CIH1D, CIH7D, and the respective normoxic control Nx7D. For short-term CIH (pre-HTN) at day 14, the CIH14D included *n* = 6 and the controls Nx14D, *n* = 5, the CIH21D, *n* = 6 and the controls Nx21D, *n* = 11. For long-term CIH (established HTN) CIH35D, *n* = 8; Nx35D, *n* = 6; CIH60D, *n* = 6; and Nx60D, *n* = 6.

It is well established that up to 7 days, there is no impact of CIH on BP, animals’ weight gain [26,34], or oxygen partial pressure at the kidney [35]. For these reasons and in compliance with the 3Rs policy of animal experimentation, the same group of animals (Nx7D) was used as normoxic controls for days 1 and 7 of CIH. In order to fulfill our aims, a higher number of animals were included in pre-HTN (14 to 21 days) and established HTN/long-term CIH (35 to 60 days) timepoints [26,34]. All rats survived independently on the duration of CIH. Body weight (BW), water, and food intake were recorded once a week together with urine sampling at the end of each rat’s active period. At the last day of each condition, the rats were anesthetized by intraperitoneal injection with medetomidine (0.5 mg/kg BW; Domitor^®^, Pfizer Animal Health, Auckland, New Zealand) and ketamine (75 mg/kg BW; Imalgene 1000^®^, Mérial, Lyon, France). Death was confirmed by cervical dislocation before tissue collection. The left kidney was collected for histology and the right kidney was dissected to obtain the kidney cortex. Approximately 120 mg of tissue was used for thiol quantification.

The measurement of invasive markers at the renal cortex required the animals to be sacrificed at different ages and with different BWs. Also, the large number of animals used made it difficult to use only littermates. Moreover, for the different study timepoints, the analytical quantification of target molecules was performed on different days. As such, to obtain a temporal overview of the impact of CIH on the evaluated markers, a Nx control group was needed for each timepoint to correct for sources of variation.

To validate that AhR activation is responsible for increasing CYP1A1 levels, we used frozen tissues from a previous study where we showed that the antagonist of AhR (CH-223191) was able to revert CIH-induced HTN [26]. Briefly, the rats (*n* = 11) were submitted to 21 days of CIH to allow the establishment of the HTN. Then animals were divided in 2 groups: group 1 (*n* = 5)—rats were treated for 14 days with CH-223191, in a dose of 5 mg/kg in 1 mL of vegetable oil (once a day, by oral gavage), simultaneously with CIH; group 2 (*n* = 6)—vehicle, rats received 1 mL of vegetable oil for 14 days simultaneously with CIH. At the end of the protocol, the rats were sacrificed as described above. The right kidney was dissected to obtain the kidney cortex for Western-blot analysis.

#### 2.2.2. Chronic Intermittent Hypoxia

A CIH paradigm already validated by our team was used [7,26,34]. Briefly, the rats were kept in an eucapnic atmosphere inside medium A-chambers (Biospherix Ltd., New York, NY, USA) and submitted to IH for 10.5 h per day. The chambers were equipped with gas injectors and O_2_ and CO_2_ sensors levels to ensure the accuracy of CIH cycles. The oxygen concentration inside the chambers was regulated by electronically regulated solenoid switches that controlled 100% N_2_ and 100% O_2_ gas input in a three-channel gas mixer and gradually lowered the oxygen in the chamber from 21% to 5% O_2_ (OxyCycler AT series, Biospherix Ltd.). The chambers were infused with 100% N_2_ for 3.5 min to briefly reduce the O_2_ concentration to 5%; afterwards, the chambers were infused with 100% O_2_ for 7 min to restore O_2_ to ambient levels of 21% until the start of the next CIH cycle. Each CIH cycle had a duration of 10.5 min (5.6 CIH cycles/h) and the CIH period was during the rats’ sleep period (inactive phase/lights on). During the remaining hours of the day, the chambers were ventilated with a constant flow of room air (21% of O_2_). Control rats were kept in the same room under Nx (21% O_2_ and 79% N_2_) for 7, 14, 21, 35 and 60 days.

### 2.3. Kidney Parameters

#### 2.3.1. Renal Histology

Histological analysis was performed in 10% formaldehyde-fixed paraffin-embedded left kidney for the rats submitted to 60 days of CIH and respective Nx controls. Sections from three rats per group were stained with hematoxylin and eosin (H&E).

#### 2.3.2. Kidney Weight and Urinary Albumin-to-Creatinine Ratio

The kidneys (right and left) were weighted for both Nx35D and CIH35D groups.

The urinary albumin-to-creatinine ratio (uACR) was obtained by dividing albumin levels by creatinine levels (uCr) in a spot urine sample.

The levels of uCr were quantified by a methodology adapted from George and collaborators (2006) [36]. Briefly, after centrifugation (800× *g*, 5 min, 4 °C), the urine samples were diluted (1:20 to 1:40) in reverse osmosis water. A total volume of 20 µL was analyzed by high-performance liquid chromatography with ultraviolet detection (HPLC-UV) at 220 nm on an Agilent 1100 Series equipment (Agilent Technologies, Santa Clara, CA, USA) using a reversed-phase Luna C18 (250 mm × 4.6 mm; 5 µm; 100 Å; Phenomenex, Torrance, CA, USA) in a column oven at 25 °C. Cr (retention time of 4.3 min) was separated in an isocratic elution mode, with a flow rate of 1 mL/min, using a mobile phase of 10 mM potassium dihydrogen phosphate solution (pH 4.7).

Albuminuria levels were measured using the Bromocresol Green Albumin (BCG) Assay Kit (MAK124, Sigma-Aldrich). The assay was performed in duplicate following the manufacturer’s instructions in a 96-well flat bottom plate. First, 5 µL of urine samples or standards were transferred into each well followed by the addition of 200 µL of BCG reagent. After an incubation period of 5 min at room temperature, the absorbance was measured at 620 nm using a microplate reader (Biotrack II plate reader, Amersham Biosciences, Amersham, UK).

### 2.4. Western-Blot Analysis

Kidney cortex samples were homogenized with 300 μL of ice-cold lysis buffer solution (Tris pH 7.4; EDTA pH 8.0; NaF; Na_3_VO_4_; Na_4_P_2_O_7_ and triton) with a cocktail of protease inhibitors using a sonifier (Branson Sonifier SFX 150). This procedure was performed on ice. The homogenates were then centrifuged for 14 min at 13,000× *g*, 4 °C. The supernatant was recovered, and its protein content was quantified using a BCA Protein Assay kit following the manufacturer’s instructions.

Protein samples (20 μg) were diluted with loading buffer, boiled, and loaded in 10% SDS-polyacrylamide gel and transferred to nitrocellulose membranes using a Trans-Blot Turbo Transfer System (Bio-Rad Laboratories, Hercules, CA, USA), at 25 V and 2.5 A for 28 min. The membranes were blocked with a solution of 5% BSA in wash buffer (0.1% Tween-20 in TBS) for at least 1.5 h at room temperature. Thereafter, the blocking solution was removed, and the blots were rinsed three times in the wash buffer. The membranes were incubated with primary antibodies against CYP1A1 (overnight) and β-actin (40 min). Then, the membranes were washed with TBST buffer (0.1%) before being incubated with mouse anti-rabbit (1.5 h) or anti-mouse (50 min) secondary antibodies for CYP1A1 and β-actin, respectively. After the membranes were washed three times with TBST buffer, they were developed in a Chemidoc Touch Imaging System (Bio-Rad Laboratories), using an ECL kit. Signal quantification was performed using the ImageLab Software system version 6.0.1.34 (Bio-Rad Laboratories). β-actin was used as an internal control to normalize sample loading on the gels.

Considering the differences in age and BW gain at each timepoint and since the purpose of this experiment was to disclose the influence of the chronicity of intermittent hypoxia on CYP1A1 expression, we normalized the values of CIH groups to those of the respective controls in Nx, considering the Nx groups as 1 (or 100%).

### 2.5. Quantification of Cysteine-Related Thiolomic Profile

The cysteine-related thiolomic profile was obtained through the quantification of cysteine (Cys), glutathione (GSH), and cysteinylglycine (CysGly) fractions: total, free total, and free reduced in the kidney cortex. The total fraction of each thiol represents the sum of free reduced (RSH) + free oxidized (RSSR) + protein-bound pools (RSSP). The free total fraction represents the sum of RSH + RSSR of each thiol. Thiols were quantified by HPLC with fluorescence detection (HPLC-FD, Shimadzu Scientific Instruments Inc., Columbia, MD, USA), after sample pre-treatment for the separation of the different pools, as previously described [7,20].

For sample pre-treatment, approximately 120 mg of kidney cortex were homogenized in 400 μL of iced PBS 1×. After centrifugation, the supernatant was collected for total thiol fraction (50 µL) and for free total and reduced fractions (350 µL) measurements. The total thiol fraction was obtained by reducing the sulfhydryl groups with TCEP (100 g/L, 5 μL). After 30 min of incubation at room temperature, the samples were treated with TCA (100 g/L containing 1 mM EDTA, 45 μL) for protein precipitation. The mixture was then vortexed and centrifuged (13,000× *g*, 10 min, 4 °C) and the supernatant collected (25 μL) to a new tube, containing NaOH (1.55 M, 5 μL), Na₂B₄O₇·10H₂O (pH 9.5, 125 mM with 4 mM EDTA, 62.5 μL), and SBD-F (1 g/L in Na_2_B_4_O_7_ buffer, 25 μL). The final mixture was vortexed and incubated in the dark, at 60 °C for 1 h, to complete the derivatization of the free sulfhydryl groups. For the free total (RSSR + RSH) and the reduced (RSH) thiol fractions, 350 μL of tissue supernatant were added to an Amicon 10K filter (UFC801024, Amicon^®^ Ultra-4 Centrifugal Filter Unit, Merck) and centrifuged (3800× *g*, 1 h, 4 °C). After this, two aliquots of 50 μL were submitted to protein precipitation with TCA, with subsequent centrifugation (13,000× *g*, 10 min, at 4 °C). Then, while one aliquot was reduced with the TCEP reagent for total non-protein bound fraction quantification, the other was incubated with reverse osmosis water to obtain the naturally reduced RSH fraction. After incubation at room temperature for 30 min, the same derivatization protocol described above was followed.

For the HPLC analyses, a reversed-Phase C18 LiChroCART 250-4 column (LiChrospher 100 RP-18, 5 µm, VWR) was used, in a column oven at 29 °C on isocratic elution mode for 20 min, at a flow rate of 0.8 mL/min. The mobile phase consisted of 100 mM acetate buffer (pH 4.5) and methanol (99:1 (*v/v*)). The detection was performed with RF 10AXL fluorescence detector, operating at excitation and emission wavelengths of 385 and 515 nm, respectively.

The measured thiol levels were normalized for initial tissue weight (approx. 120 mg) and BW. The protein-bound fraction (RSSP) was obtained by subtracting the total free fraction from the total fraction. This procedure has been validated by others [14] and is well established in several publications [7,20,37,38]. The free oxidized fraction (RSSR) was obtained by subtracting the total free reduced to the total free fraction (RSH). In case of concentrations below the limit of quantification, values were represented by the square number of the limit of quantification.

### 2.6. Cell Culture

Human umbilical vein endothelial cells (HUVECs: ATCC^®^ CRL-1730™) were obtained from American Type Culture Collection (ATCC) and cultured in Endothelial Cell Growth Basal Medium-2 (EBM-2: CC-3156, Lonza Bioscience, Basel, Switzerland) supplemented with EGM-2 SingleQuots Supplements (CC-4176, Lonza Bioscience), at 37 °C in a humidified environment of 5% CO_2_, according to previous experience [39]. HUVECs (2 × 10^5^ cells/well) were plated in 6-well plates and exposed to cysteine (0.2, 0.4 and 0.8 mM) and cystine (0.1, 0.2 and 0.4 mM) for 6 and 24 h [40,41,42].

### 2.7. Quantitative Real-Time PCR

RNA was extracted using RNeasy Mini Extraction kit (74104, Qiagen, Germantown, MD, USA) and cDNA synthesized from 1 µg RNA, using SuperScript II Reverse Transcriptase (18080e44, Invitrogen, Waltham, MA, USA), both according to the manufacturer’s protocol. Quantitative Real-Time PCR was performed using Power SYBR Green PCR Master Mix (4367659, Applied Biosystems, Waltham, MA, USA), according to the manufacturer’s protocol. The reaction was carried out in a LightCycler^®^ 480 instrument (Roche). Primers used were AHR (Fwd: GGTCTCCCCCAGACAGTAG; Rev: CCCTTGGAAATTCATTGCCAG); CYP1A1 (Fwd: CTGGGTTTGACACAGTCACAAC; Rev: GCAGATGGGATCTGTCAGAG); HIF1α (Fwd: GCAGCAACGACACAGAAACTG; Rev: GTGGGTAATGGAGACATTGCC) and xCT (Fwd: GGTCCTGTCACTATTTGGAGC; Rev: GAGGAGTTCCACCCAGACTC). Hypoxanthine-guanine phosphoribosyltransferase (HPRT; Fwd: TGACACTGGCAAAACAATGCA; Rev: GGTCCTTTTCACCAGCAAGCT) was used as a housekeeping gene.

### 2.8. Data Analysis

#### 2.8.1. Univariate Analysis

Data are presented as the mean ± standard error of the mean (SEM). The impact of IH chronicity in the different endpoints was analyzed using GraphPad Prism software version 8 (GraphPad Software, San Diego, CA, USA) and a *p*-value < 0.05 was set to consider statistically significant differences.

#### 2.8.2. Multivariate Analysis

Multivariate analyses were performed to assess the temporal changes of IH in the cysteine-related thiolomic profile. The dataset consisted of 11 fractions (total, free total, free oxidized, free reduced, and protein-bound Cys; total, free total, and protein-bound CysGly; total, free total, and protein-bound GSH) × 74 observations (rats). Data was mean-centered and scaled to unit variance before statistical analysis. Principal Component Analyses (PCA) and Geometric Trajectory Analysis [43] were performed to evaluate the influence of time and condition (Nx or CIH) on the thiolome. Due to the design of the study (rats are different at each time point), Partial Least Square Discriminant Analysis (PLS-DA) was performed at each time point to disclose the most relevant metabolites discriminating Nx and CIH. Both PCA and Geometric Trajectory Analysis were performed with R environment (http://www.r-project.org/ R version 3.6.1, accessed on 2 July 2021), using the “*Rcpm*” and “*pcaMethods*” packages. Visualizations were made using “*ggplot2*” and “*gridExtra*” packages. PLS-DA models were performed with SIMCA software (MKS Umetrics, Umeå, Sweden Umetrics, version 16.0.1).

## 3. Results

### 3.1. Impact of Intermittent Hypoxia Chronicity in Animals Body Weight, Food and Water Intake

**Figure 1:**
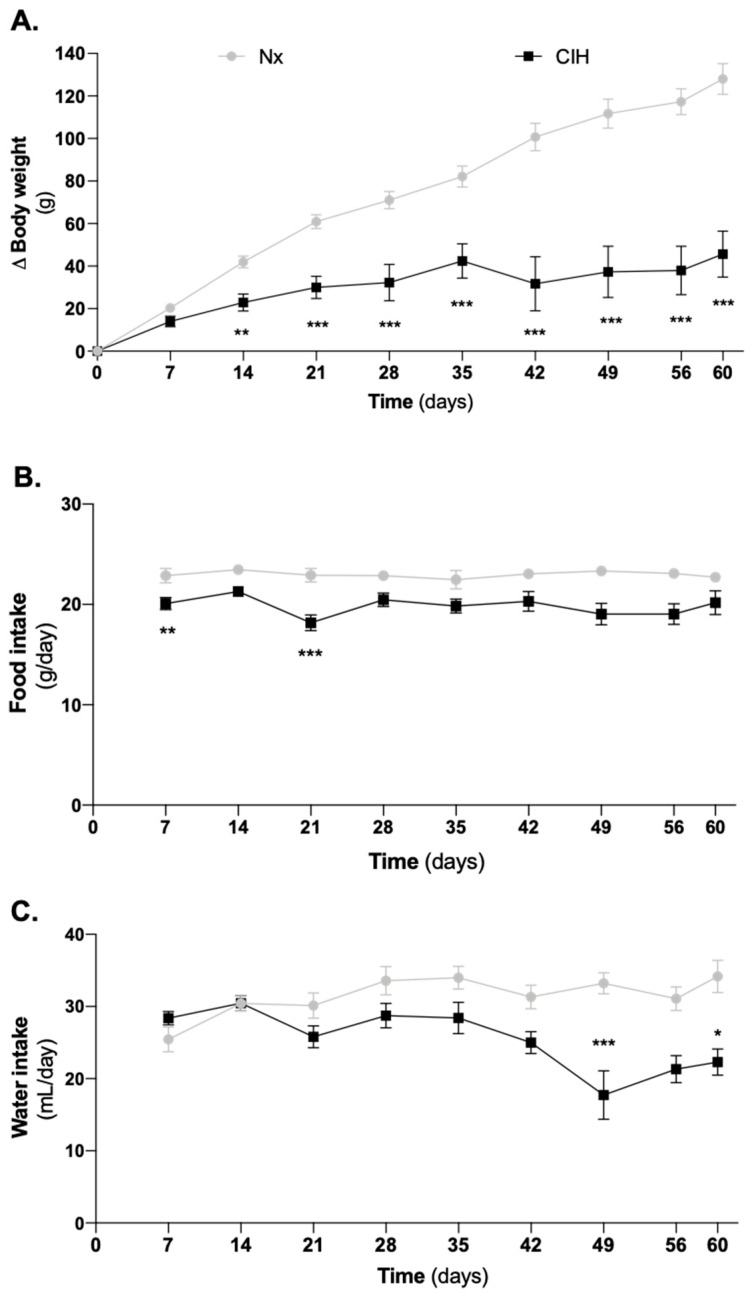
Impact of CIH on (**A**) the animals’ growth curves for body weight, (**B**) food intake, and (**C**) water intake. The CIH animals showed (**A**) a disruption of the growth curve for body weight, (**B**) a slight decrease in food consumption particularly in the period of HTN development, and (**C**) a decrease in drink consumption in long-term CIH. Data are presented as variation of body weight from baseline. Two-way ANOVA with Bonferroni’s multiple comparison test; comparison Nx vs. CIH: * *p* < 0.05; ** *p* < 0.01; *** *p* < 0.001. Number of animals per group: Day of study (*n* for Nx; *n* for CIH): D7 (33; 31), D14 (25; 26), D21 (20; 20), D28 (12; 14), D35 (12; 14) and D42 to D60 (6; 6). CIH: chronic intermittent hypoxia; Nx: normoxia.

Nx and CIH age-matched (8–17 weeks) groups had similar baseline BW (275 ± 11 g vs. 266 ± 13 g, Nx vs. CIH). However, CIH groups had a lower BW gain than Nx groups (Figure 1A). This was evident right after 14 days of CIH and it was maintained over time. Whereas short-term CIH decreased food intake (Figure 1B), water intake (Figure 1C) was only affected after established HTN.

### 3.2. Impact of Intermittent Hypoxia Chronicity in Kidney Parameters

**Figure 2:**
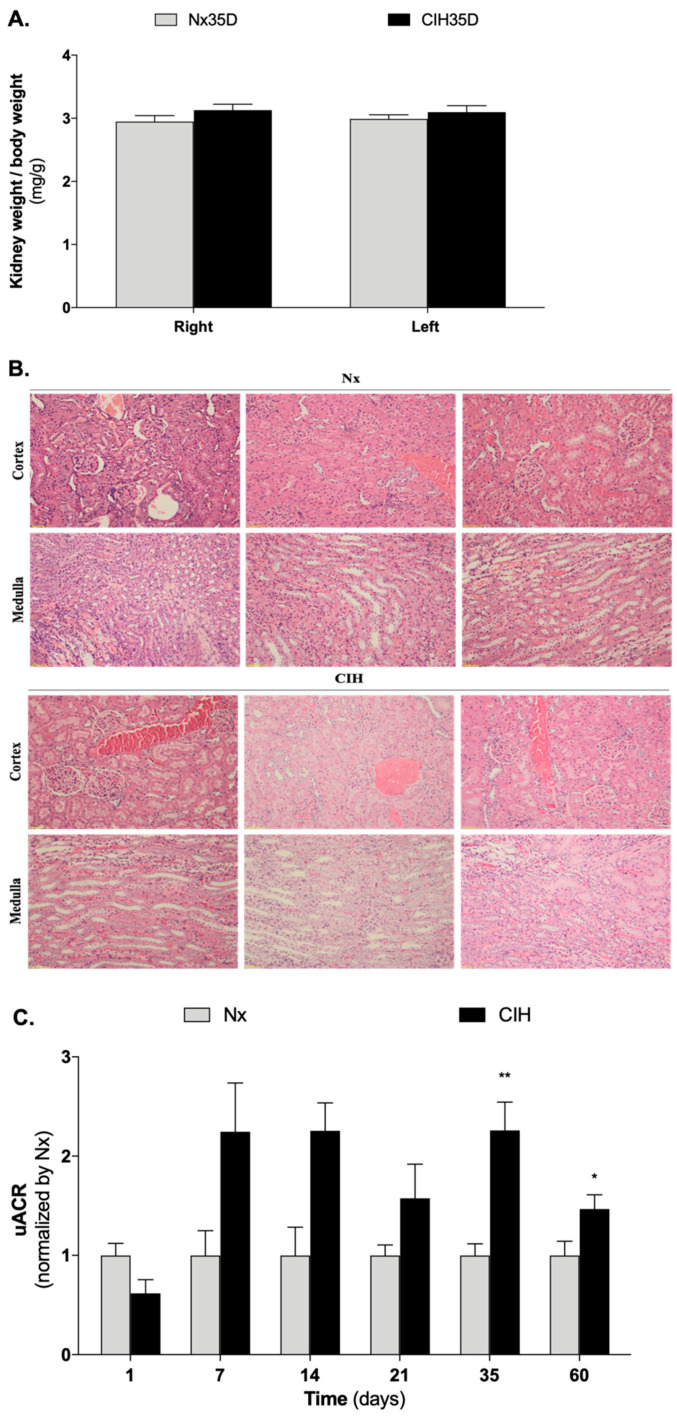
Impact of CIH on (**A**) kidney weight, (**B**) kidney histology, and (**C**) uACR. (**A**) No differences were found in the ratio of kidney weight to body weight (*n* = 6 for Nx35D; *n* = 8 for CIH35D). (**B**) No relevant histological changes were observed for the left kidney of all groups (*n* = 3 animals per group). Staining was performed in 10% formaldehyde-fixed paraffin-embedded left kidney sections using H&E; Magnification: 10×; (**C**) CIH increases uACR. Number of animals per group: Day of study (*n* for Nx; *n* for CIH): D1 (8; 4), D7 (4; 4) D14 (4; 2) D21 (6; 8); D35 (6; 8); D60 (9; 7). Multiple *t*-test; comparison Nx vs. CIH: * *p* < 0.05; ** *p* < 0.01. CIH: chronic intermittent hypoxia; Nx: normoxic; uACR: urinary albumin-to-creatinine ratio.

No changes in right (Nx vs. CIH: 1.12 g vs. 1.06 g) and left (Nx vs. CIH: 1.13 g vs. 1.04 g) kidney weight were found at D35 (data not shown), even when the data was normalized for BW (Figure 2A). In addition, no relevant histological changes were found at the end of the study (Figure 2B). Nevertheless, uACR was increased in the CIH group (Figure 2C).

This represents the first longitudinal assessment of uACR in this CIH-HTN model. While large (more than two-fold) apparent changes in the uACR were observed, they did not reach statistical significance at days 7 and 14. It is possible that the experiments were statistically underpowered at these timepoints. Thus, while long-term CIH definitely increased uACR, short-term IH may also have a similar effect.

### 3.3. Impact of the Chronicity of Intermittent Hypoxia in AhR Activation at Kidney Cortex

**Figure 3:**
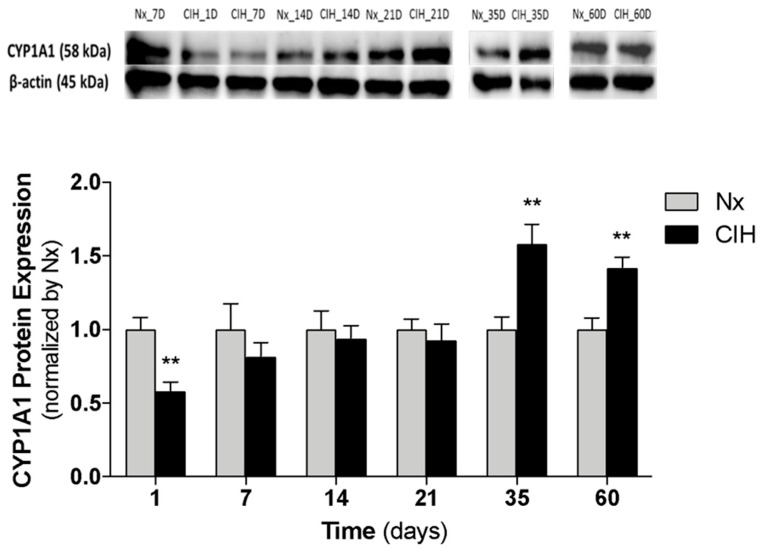
Impact of chronicity of IH in CYP1A1 protein expression at kidney cortex. CYP1A1 protein levels were quantified in the renal cortex of rats exposed to 1, 7, 14, 21, 35 and 60 days of CIH and Nx (controls); *n* = 5 animals per group. Nx7D was used as control for CIH1D and CIH7D. Multiple t-test; comparison Nx vs. CIH: ** *p* < 0.01. CIH: chronic intermittent hypoxia; CYP1A1: cytochrome P450 family 1, subfamily A, polypeptide 1; Nx: normoxia.

To understand the impact of IH chronicity in AhR activation, we assessed CYP1A1 protein expression (Western-blot) in the kidney cortex of animals exposed to different durations of IH (Figure 3). CYP1A1 expression decreased at day 1 of CIH (*p* = 0.003) and progressively increased, reaching near Nx levels between 14 and 21 days (established HTN). After long-term CIH exposure (35 and 60 days), CYP1A1 expression was higher in CIH than in Nx controls (Figure 3).

**Figure 4:**
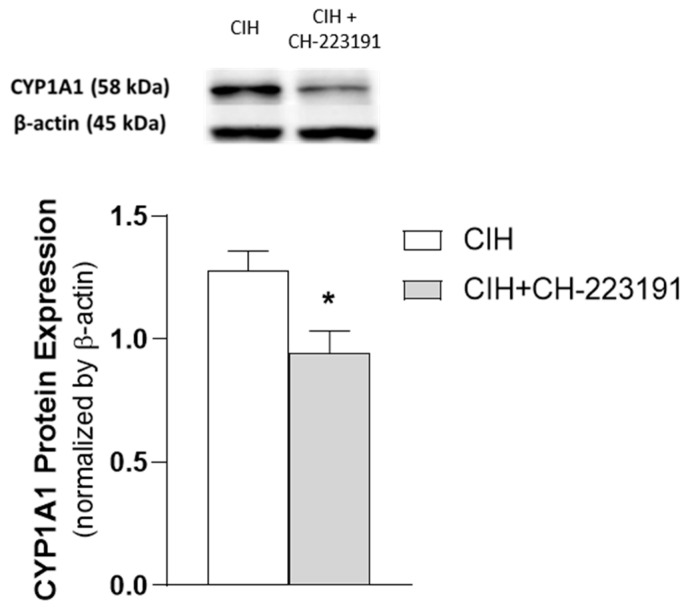
Effect of CH-223191 in CYP1A1 protein expression in renal cortex tissue of CIH-exposed rats (35 days). CYP1A1 protein levels were quantified in the renal cortex of CIH-exposed rats administered with CH-223191 (*n* = 5) or vegetable oil (CIH) (*n* = 6). Mann–Whitney’s U-test; comparison CIH vs. CIH + CH-223191: * *p* < 0.05. CYP1A1: cytochrome P450 family 1, subfamily A, polypeptide 1.

Additionally, the observed increase in CYP1A1 protein levels induced by long-term CIH exposure (35 days) was reverted by the administration of the AhR antagonist CH-223191 (Figure 4).

### 3.4. Cysteine-Related Thiolomic Profile

We herein assessed the effect of short- and long-term IH in the cysteine-related thiolomic profile. The concentrations of CysGly and GSH free total pool were below the resolution limit in 30 and 74% of samples, respectively.

**Figure 5:**
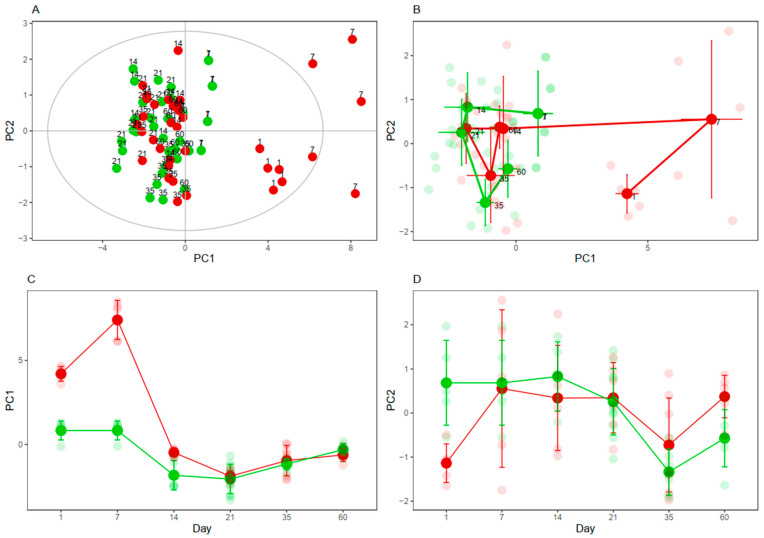
Impact of CIH on cysteine-related thiols. (**A**) Principal Component Analysis performed with the 11 thiol fractions and 74 observations; the first and second components explained 63 and 11% of the variance of the data, respectively. (**B**) Geometric Trajectory Analysis showing PC1 vs. PC2. (**C**) Geometric Trajectory Analysis showing PC1 vs. time. (**D**) Geometric Trajectory Analysis showing PC2 vs. time. Rats submitted to Nx are colored in green and rats submitted to CIH are colored in red. Smaller and lighter dots represent individual rats; bigger and more intense dots represent the average values at (**B**–**D**). Nx7D was used as control for CIH1D and CIH7D. PC: Principal Component.

The PCA is a multivariate analysis commonly used to assess the main sources in the variance of the data. Herein, the PCA was performed to evaluate if CIH influenced the concentrations of the cysteine-related thiols. A useful representation of the PCA is the score plot, which represents the samples (rats) according to the first two components of the PCA model. The first two components are new variables built from the 11 cysteine-related thiol fractions and explain the most and second most variance of the data, respectively. In our model, the first and second components explained 63 and 11% of the variance of the data, respectively. The influence of CIH was clear at 1, 7, and 14 days, wherein the CIH animals appeared on the right side and Nx controls on the left side of the plot (the greater the distance among samples, the more different they are). The differences found in longer periods were less clear (Figure 5A).

To better visualize the time-dependent effects of CIH on the cysteine-related thiols, geometric trajectory analysis was performed. In this analysis, the average of the principal components 1 and 2 of the rats belonging to each condition (Nx and CIH) and timepoint (1, 7, 14, 21, 35, and 60) are calculated and plotted in the score plot (Figure 5B) or versus time (Figure 5C,D). According to this trajectory analysis, the major differences found in the cysteine-related thiolomic profile between Nx and CIH occurred mainly up to the 14-day timepoint (pre-HTN).

**Figure 6:**
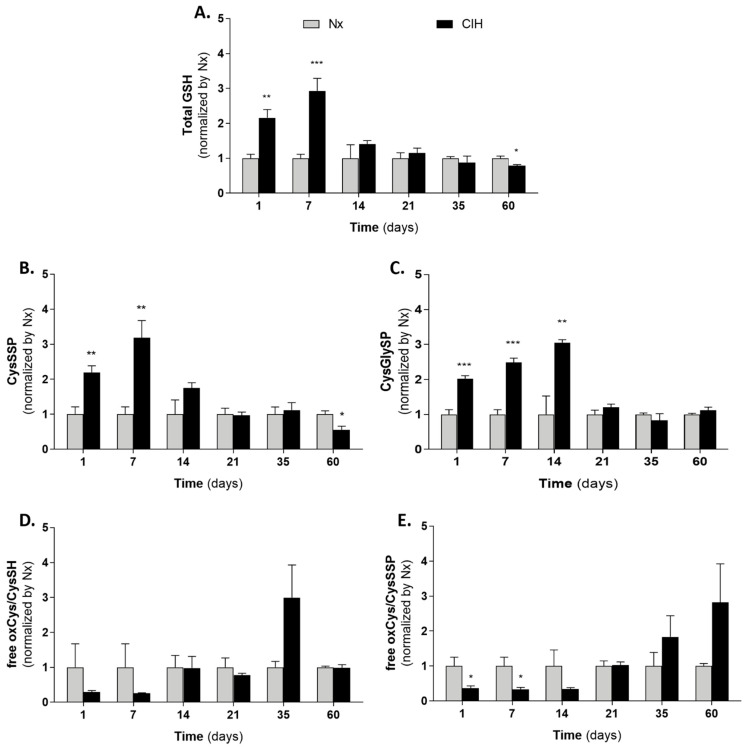
Impact of IH chronicity cysteine related thiolome in kidney cortex. (**A**) Total glutathione; (**B**) Cysteinylated proteins; (**C**) Cysteinylglycinylated proteins; (**D**) free oxidized Cys to reduced Cys ratio; (**E**) free oxidized Cys to Cysteinylated proteins ratio. Nx7D was used as control for CIH1D and CIH7D. Number of animals per group: Day of study (*n* for Nx; *n* for CIH): D1 (5;5), D7 (5; 5) D14 (5; 6) D21 (11; 6), D35 (6; 8) and D60 (6; 6). Multiple *t*-test; comparison Nx vs. CIH: * *p* < 0.05; ** *p* < 0.01; *** *p* < 0.001. CIH: chronic intermittent hypoxia; Cys: cysteine; CysGly: cysteinylglycine; CysGlySP: cysteinylglycinylated proteins; CysSH: reduced cysteine; CysSSP: cysteinylated proteins; GSH: glutathione; Nx: normoxia; oxCys: oxidized cysteine.

PLS-DA models were then performed at days 1, 7, and 14 to identify the most relevant thiols affected by CIH. Those thiol fractions with a variable importance on the projection (VIP) value > 1 were selected as the most relevant metabolites discriminating Nx from CIH, which were analyzed individually by multiple t-test. Short-term IH increased total GSH availability that gradually decreased until the end of the study (Figure 6A). This was similar to the pattern found for protein *S*-cysteinylation (CysSSP) (Figure 6B), but contrary to the one for CYP1A1 protein expression (Figure 3). Both GSH and CysSSP decreased from day 14 differently from CysGlySP, which increased until day 14 and was never lower in CIH than in Nx (Figure 6C). On the contrary, free oxidized cysteine had a similar profile to CYP1A1 (Figure 6D,E): it decreased in short-term CIH and increased with long-term CIH. While not reaching statistical significance, there is a tendency for free cysteine to change from a reduced to a more oxidative status in long-term CIH (Figure 6D). Moreover, oxidized cysteine exists mainly extracellularly and comprises two dynamic pools, the free oxidized and the CysSSP. Both pools might represent a way to supply cysteine into the cell [7,16,38,44], so we also analyzed the ratio between them. We observed that longer periods of CIH favors increased free oxidized cysteine instead of CysSSP pool (Figure 6E). At day 35, when CYP1A1 was increased, the levels of oxidized cysteine in CIH and Nx groups were 6.95 ± 0.91 and 3.69 ± 0.54 µM/g of tissue/g of BW, respectively (Mann–Whitney’s U-test; *p* = 0.0127) (data not shown).

### 3.5. Effect of Cysteine and Cystine on AhR Canonical Pathway Activation

To address the effect of cysteine and cystine on both AhR and its downstream target gene CYP1A1, gene expression was evaluated in HUVEC cells exposed to a range of Cys and cystine concentrations using early (6 h) and late (24 h) timepoints. Both AhR [45] (reviewed in [46]) and oxidized cysteine [47,48] (reviewed in [31]) have been described to impact endothelial cells, promoting toxicity to the cardiovascular system. This knowledge supported the selection of HUVECs for this proof-of-concept study of the interplay between cysteine redox changes and AhR.

**Figure 7:**
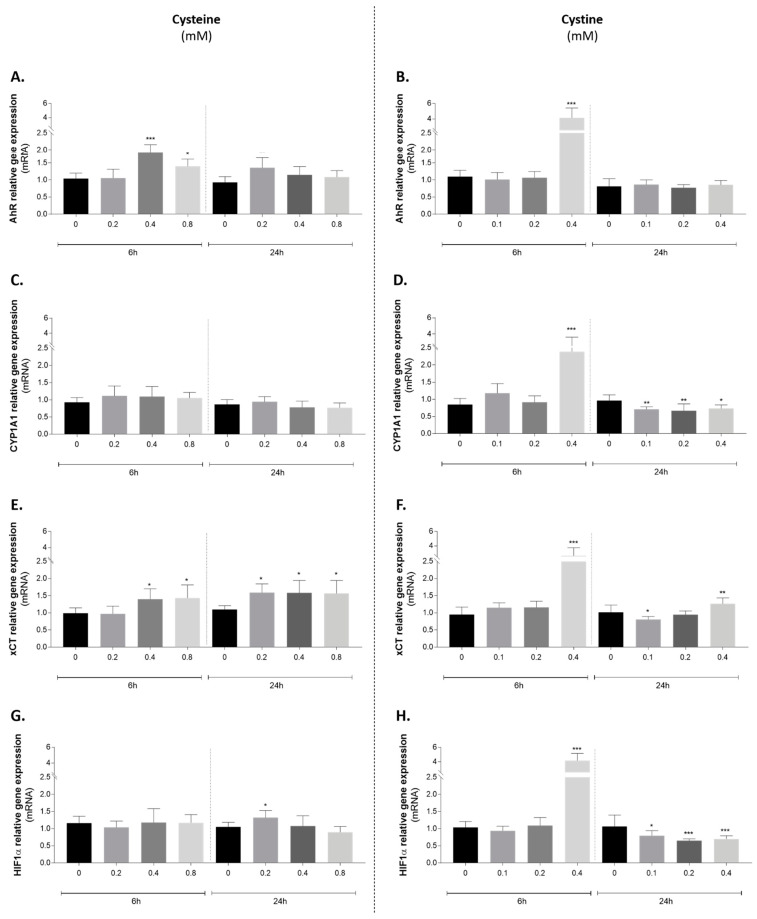
Effect of cysteine (left panel) and cystine (right panel) administration to endothelial cells on the expression of (**A**,**B**) *Ahr*; (**C**,**D**) *Cyp1a1*; (**E**,**F**) xCT and (**G**,**H**) *Hif1α*. One-way ANOVA with Dunnet’s multiple comparisons test vs. control condition: * *p* < 0.05; ** *p* < 0.01; *** *p* < 0.001. AhR: aryl hydrocarbon receptor; CYP1A1: cytochrome P450 family 1, subfamily A, polypeptide 1; Hif1α: hypoxia inducible factor 1α; xCT: cysteine/glutamate transporter.

There was a clear difference between the two stimuli (Figure 7, left-cysteine A, C, E, G vs. right cystine B, D, F, H). Although we cannot exclude a contribution of cystine for cysteine effects (i.e., from cysteine spontaneous oxidation in media or the content of cystine in the media), cysteine administration produced no or mild increases in the expression of all genes investigated (Figure 7A,C,E,G). At equimolar concentrations (0.4 mM) and comparatively to cysteine, cystine treatment was a stronger inducer of *Ahr* expression and promoted AhR activation, as indicated by *Cyp1a1* increase (Figure 7B,D). In addition, for *Cyp1a1*, there was a clear difference between the earlier and later effects, which can be related to cyst(e)ine metabolism. Additionally, the expression of the glutamate/cystine antiporter (xCT), responsible for the import of cystine, but not of cysteine, was also mostly increased by cystine as an earlier effect (Figure 7F). As HIF1α is a hypoxia-responsive regulator and it shares the same binding partner of AhR (HIF1β also known as ARNT), its expression levels were also investigated (Figure 7H). The highest cystine concentration induced a positive effect in the earlier timepoint, but a repression of *Hif1α* was detected at 24 h in all tested concentrations (Figure 7H).

To sum up, we investigated the impact of CIH in the following endpoints: BW, food and water intake, kidney status, AhR activation, and cysteine-related thiolomic profile. Our data showed that the differences between Nx and CIH regarding AhR and thiols were evident in short- and long-term IH (established HTN), mostly in opposite directions. At pre-HTN, we found similar endpoints between Nx and CIH, except for food intake and CysGly protein-bound fraction. In short-term IH and comparatively to Nx, we found that CYP1A1 expression was lower, protein *S*-thiolation and total GSH were increased, and there was a reduction in food consumption. In opposition, in long-term IH, the CYP1A1 expression was increased and protein *S*-cysteinylation and GSH were decreased. Moreover, free oxidized Cys increased with the same temporal pattern of AhR activation, which led us to the in vitro study, which showed that the observed increase in oxidized Cys might promote AhR in endothelial cells. In addition, long-term IH decreased water intake and an established increase in uACR was observed without changes in kidney weight and histology.

## 4. Discussion

In this work, we have investigated the impact of the chronicity of IH in the kidney cortex at three phases according to HTN development: short-term (days 1 and 7), pre-HTN (days 14 and 21), and established HTN/long-term IH (days 35 and 60). Although BP increased right from the first week [26,34], we referred to the first week differently from pre-HTN, as short-term IH has been associated with cardioprotective effects [49] and reversible redox changes in antioxidant enzymes [50]. We defined the phase 14–21 days as pre-HTN because BP is still increasing in this point, and a further plateau of systolic and diastolic BP is achieved at day 35 [34].

Oxidative stress and hypoxia in the kidney are unifying paradigms of HTN [51,52,53]. OSA-HTN is not an exception; it promotes functional and structural kidney damage and increases oxidative stress, renin-angiotensin-aldosterone system, and sympathetic nervous system activity [54,55]. There are few studies dedicated to the impact of IH chronicity in kidney molecular signatures and the existing conflicting data might be due to the variable frequency, intensity, and duration of CIH paradigms used. Indeed, long- and short-term IH induce different molecular and physiological responses. Short-term IH is characterized by oxidative and inflammatory events that elicit compensatory/adaptive responses [8].

PCA and trajectory analyses showed that the thiolome was mainly affected by short-term IH. Interestingly, our results showed that acute and short-term IH suppressed the activation of AhR-CYP1A1 axis and favored protein *S*-thiolation formation and increased GSH availability at the kidney cortex, instead of free oxidized Cys. This increase in post-translational modification, protein *S*-thiolation, may protect proteins from irreversible oxidation [56], constituting a mechanism of redox switch in endothelial cells that accounts for vascular homeostasis [57] and serving as a sensitive biomarker for the extent of oxidative stress [17].

Protein *S*-cysteinylation has been described as part of the redox signaling mechanism of the “disulfide stress phenomena” described in acute organ inflammation [58]. The targets of this disulfide stress have important roles in DNA repair, cell proliferation, apoptosis, endoplasmic reticulum (ER) stress, inflammatory response [58], and induction of adaptive responses through the activation of both the unfolded protein response [59] and the antioxidant response element [60,61,62]. These mechanisms were corroborated by Sun and collaborators (2012), who showed a protective response against kidney oxidative damage (increased renal Nrf2, hemeoxygenase-1 (HO-1) and metallothionein (MT), and decreased malondialdehyde (MDA)), using a short-term (3–7 days) moderate-to-severe IH paradigm [5]. Interestingly, acute low doses of IH (1–2 h/day) have been associated with a therapeutic benefit in BP control [63], without prompting CIH comorbidities, and have shown to be safe for the common adverse drug reactions of antihypertensive drugs [49].

Differently, the chronicity of IH induces de-compensatory/maladaptive responses [8]. Long-term moderate-to-severe IH (20 to 60 cycles/h. 21–8% O_2_, 35–60 days) induces kidney histological changes [64,65]; increases markers of fibrosis [5,6,65], inflammation [5,6,66,67], oxidative stress [3,5,6,66], and ER stress [3]; promotes abnormal mitochondrial dynamics [68] and cell apoptosis [3,5,6,64,67]; increases growth factors [64], noradrenaline [65] and Ang II concentrations [6]; stabilizes Hif1α [5] and increases serum Cr and blood urea nitrogen (BUN) levels [67] and albuminuria [64]. It is important to note that the aforementioned studies were performed in long-term moderate and severe IH rodent models. This might explain the absence of histological changes in our study that relies on a mild paradigm. Even severe IH paradigms (45 cycles/h, 21–9% O_2_) for 14 days, only caused mild renal morphological damage (hyperplasia of glomerular mesangial cells), which was aggravated with longer CIH exposure (28 days) [69].

This suggests that in this model, HTN precedes kidney damage. Similar observations were made by the Almarabeh team [70], which showed that mild IH for 14 days did not affect baroreflex control of renal sympathetic nerve activity. Although both diuretic and natriuretic responses were impaired [70], AhR and cystine may not underlie these mechanisms because at this timepoint (2 weeks, pre-HTN), those two parameters were not affected by CIH.

We found no histological or kidney weight changes in established HTN, but there was an increase in ACR. Although no apparent renal histological damage was observed, our current and previous pre-clinical data support that CIH induces some degree of kidney dysfunction through the increase in uACR, increased expression of renal fibronectin at day 21 and 60 of CIH [7], and inflammatory and epithelial to mesenchymal transition markers at day 21 [26].

In OSA patients, albuminuria has been related to an increased apnea-hypopnea index [71] and the ACR ratio is higher in OSA patients with HTN than hypertensive patients without OSA [72]. ACR, AhR, and CYP1A1 have been related with HTN in clinical genome-wide association studies (GWAS) [73,74]. The AhR-CYP1A1 axis has also been associated with HTN in epidemiological and in vivo studies (reviewed in [27]). However, the exact contribution of CYP1A1 to increased BP is yet to be identified. For instance, CYP1A1 metabolizes melatonin [75], which is known to decrease renal oxidative stress and vascular reactivity [76] and to have anti-hypertensive effects [77,78].

Our in vivo data showed that AhR-CYP1A1 activation at established HTN occurs concomitantly with an accumulation of free oxidized Cys, the major non-protein bound form of Cys extracellularly (mainly cystine) [15], suggesting a putative link between cystine and AhR activation. The in vitro study in HUVECs reinforces this result.

Recently, Lopes-Coelho (2021) showed that the angiogenic process may rely on a ferroptosis-like mechanism in endothelial cells, promoted by the blockage of xCT-related cystine cellular uptake [39]. Cystine avoids ferroptosis and increases glutathione availability, which avoids the angiogenic switch and promotes vessel stabilization. Both processes are pivotal for the prevention of vascular remodeling [79].

This question might rely on the cystine dose. Endothelial cells, when exposed to an environment with oxidized redox potential of Cys/CySS (cysteine-to-cystine ratio), presented an increased expression of NF-kB activation along with adhesion molecules [47], promoting the adhesion of monocytes to the vasculature, vascular inflammation, tissue remodeling, and (mal)adaptation [80].

In fact, higher plasma oxidized cysteine is a biomarker of endothelial dysfunction and HTN in non-OSA [81] and OSA patients [82]. Moreover, with higher doses of cystine we observed an increase in AhR expression and activation (CYP1A1) and there is strong evidence on the role of AhR activation by exogenous and endogenous metabolites in endothelial dysfunction in vitro [83,84,85,86,87] and in vivo [86,88,89].

The way how AhR is activated by cystine administration, if directly by post-translational modifications or by the action of its catabolism products (taurine, H_2_S, CoA) [16,38] requires further clarification. At the kidney level, the best-known AhR activators are uremic toxins [90], particularly protein-bound uremic toxins [91], like p-cresol sulfate and indoxyl sulfate [92,93]. Cyst(e)ine is also a protein-bound uremic toxin [94]. Those toxins may activate AhR to induce the expression of enzymes and transporters that are responsible for their reabsorption/excretion [92,95]. It would be plausible that cystine would activate AhR to increase the expression of its importer xCT (glutamate/cystine antiporter), responsible for the import of cystine (oxidized), but not of cysteine (reduced), to maintain homeostatic levels; however, to the best of our knowledge, there is no evidence of AhR’s direct involvement in xCT regulation. Interestingly, the kidney cortex almost exclusively expresses an enzyme that detoxifies cysteine disulfides. This enzyme, N-acetyltransferase 8 (NAT8), is known to be associated with kidney function and BP control [18,96], although scarce information exists on its regulation. Finally, CIH-induced AhR activation and an increased expression of xenobiotic metabolic enzymes and drug transporters might impact the metabolome at the kidney, which has been recognized as a contributor for the long-term regulation of BP [97].

Regarding hypoxia, the development of kidney cortex hypoxemia in our CIH model is not an early event, as shown in a recent study. Using a rat model submitted to a mild CIH paradigm, similar to ours (10 cycles/h; 8 h/day; 21–6.5% O_2_), the authors found a lower PO_2_ in the kidney, exclusively at cortical tissue, after 14 days (pre-HTN) [35]. HIFs and AhR compete for ARNT dimerization and this interplay reciprocally interferes with HIFs and AhR transcriptional activities [27,98,99,100]. In fact, HIF might increase ARNT expression [99] and justify the late increase in AhR activation (only when enough ARNT is available).

Finally, the effects of CIH over BW have been already studied extensively, although the mechanisms are yet to be fully identified. We previously demonstrated a decrease in BW until day 35 [34], and in this study, we confirmed that it is maintained until day 60. This decrease was consistent with other CIH paradigms in both mice and rats that applied 14 days [101] and 35 days [102] of severe CIH paradigm and 35 [103,104] and 60 days of moderate CIH paradigm [105]. Weight loss has been explained by reduced food intake and locomotion, a rise in leptin levels, or an increase in metabolism and energy expenditure, all phenomena described in CIH [101,105]. Other studies reinforce an increased metabolism and energy expenditure promoted by CIH [103]. AhR has been implicated in BW regulation [106]. While the suppression of AhR activation in short-term IH might contribute to the initial decrease in BW gain, the increase in AhR activation observed during long-term IH does not revert this effect. This might indicate that other mechanisms are maintaining the reduction in BW gain. We also observed that longer periods of IH decreased the water intake concomitantly to increased AhR activation. The AhR antagonist, CH-223191, administered from day 21 to 35 of CIH, was not able to restore the normal BW growth curve, nor the reduction in food intake induced by CIH [26]. The putative involvement of AhR in the BW growth curve under CIH deserves further elucidation. On the other hand, a causal association was reported between cystine and increased BW and increased food consumption, but without changes in water consumption [107].

## 5. Conclusions

This study adds novel players for the molecular mechanisms of CIH-HTN. Long-term IH, in contrast to acute and short-term IH, over-activates AhR-CYP1A1 at the kidney, supporting AhR blockers as putative antihypertensive drugs in OSA-HTN. In addition, acute low-dose of IH might represent an innovative therapeutic strategy for blocking AhR, and CYP1A1 might be a biomarker of OSA severity.

The chronicity of IH impacts the interplay between AhR-CYP1A1 and cysteine-related thiolome, suggesting that increased *S*-thiolation of proteins might represent a protective mechanism for CIH impact in BP and asks for innovative antioxidant mechanisms of action. The different impact of short- and long-term IH on cysteine-related thiolome highlights the importance of the use of different CIH paradigms in pharmacological studies on antioxidants. Finally, these results were obtained in a lean model of mild CIH with a low kidney compromise. This work contributes to a better understanding of the phenotype of OSA-HTN, mimicked by this model, which is in line with the major current challenges in OSA diagnostic and management strategies [108]. For instance, in this lean CIH model without histological kidney damage, upon established HTN (35 days), the nonselective β-blocker carvedilol, with intrinsic anti-α1-adrenergic and antioxidant properties [34], which is a substrate of CYP1A1 [109], was not able to reverse CIH-increased BP, which has occurred with an AhR antagonist [26].

## Data Availability

Data is contained within the article.
